# An inflammatory-related genes signature based model for prognosis prediction in breast cancer

**DOI:** 10.32604/or.2023.027972

**Published:** 2023-04-10

**Authors:** JINGYUE FU, RUI CHEN, ZHIZHENG ZHANG, JIANYI ZHAO, TIANSONG XIA

**Affiliations:** Jiangsu Breast Disease Center, The First Affiliated Hospital with Nanjing Medical University, Nanjing, China

**Keywords:** IRGs, Prognostic model, TCGA, Immune, Breast cancer

## Abstract

**Background:**

Breast cancer has become the most common malignant tumor in the world. It is vital to discover novel prognostic biomarkers despite the fact that the majority of breast cancer patients have a good prognosis because of the high heterogeneity of breast cancer, which causes the disparity in prognosis. Recently, inflammatory-related genes have been proven to play an important role in the development and progression of breast cancer, so we set out to investigate the predictive usefulness of inflammatory-related genes in breast malignancies.

**Methods:**

We assessed the connection between Inflammatory-Related Genes (IRGs) and breast cancer by studying the TCGA database. Following differential and univariate Cox regression analysis, prognosis-related differentially expressed inflammatory genes were estimated. The prognostic model was constructed through the Least Absolute Shrinkage and Selector Operation (LASSO) regression based on the IRGs. The accuracy of the prognostic model was then evaluated using the Kaplan-Meier and Receiver Operating Characteristic (ROC) curves. The nomogram model was established to predict the survival rate of breast cancer patients clinically. Based on the prognostic expression, we also looked at immune cell infiltration and the function of immune-related pathways. The CellMiner database was used to research drug sensitivity.

**Results:**

In this study, 7 IRGs were selected to construct a prognostic risk model. Further research revealed a negative relationship between the risk score and the prognosis of breast cancer patients. The ROC curve proved the accuracy of the prognostic model, and the nomogram accurately predicted survival rate. The scores of tumor-infiltrating immune cells and immune-related pathways were utilized to calculate the differences between the low- and high-risk groups, and then explored the relationship between drug susceptibility and the genes that were included in the model.

**Conclusion:**

These findings contributed to a better understanding of the function of inflammatory-related genes in breast cancer, and the prognostic risk model provides a potentially promising prognostic strategy for breast cancer.

## Introduction

Breast cancer has become the most common cancer in women and the leading cause of cancer-related deaths globally [1]. Fortunately, the number of deaths has gradually decreased due to the improvement in early diagnosis and prompt treatment of breast cancer in recent years [2–4]. At present, the choice of treatment for breast cancer depends on the stage, and primary clinical treatment includes surgical resection, chemotherapy, and radiotherapy [5,6]. Due to the great heterogeneity of breast cancer, whose etiology and pathology differ from person to person [7,8], and despite enormous advances in surgical and systematic treatment, the prognosis of breast cancer patients is not as good as expected. The prognosis of breast cancer patients is currently predicted using a variety of biomarkers [9,10]. However, there will inevitably be deviations in the relevant prediction methods. Therefore, it is crucial to establish tools that could precisely predict the prognosis of breast cancer patients and guide clinical treatment.

The relationship between inflammation and cancer is complex and varied. Earlier studies have found that inflammation plays an important role in tumor-associated illnesses [11,12]. Inflammation and cancer have a wide range of relationships that have been the subject of extensive investigation recently. Chronic inflammation linked to tumors frequently contributes to the malignant progression of tumors, promotes the advancement to a metastatic stage, and may also assist the emergence of new tumors [13,14]. Studies found that tumor-associated inflammation can speed up the tumor’s development and progression by promoting angiogenesis and metastasis, impairing anti-tumor immune responses, and altering the sensitivity of tumor cells to chemotherapeutic agents [15–17]. According to research by Kay et al., large amounts of mutagenic DNA damage frequently result from inflammation. And damaged DNA typically causes mutations. Additionally, unrepaired DNA damage brought on by inflammation promotes the development of cancer by increasing mutagenesis [14]. Also, numerous studies have shown that inflammation has been linked to breast cancer [18,19]. Recently, a large number of inflammatory cells have been found to be infiltrated in breast cancer, both in the tumor matrix and around the tumor [20]. And researchers also discovered that the levels of pro-inflammatory markers are significantly elevated in the stromal of human breast cancer as compared to normal tissues [21]. For instance, tumor necrosis factor-α (TNF-α) is a significant proinflammatory cytokine that has been found in tumor microenvironments, it is involved in all stages of breast cancer development and affects the proliferation, metastasis, and recurrence of breast cancer [22].

Although related studies have confirmed that inflammation plays an important role in the development and metastasis of breast cancer, it is still unclear whether inflammation and its related genes could affect the prognosis of breast cancer, so it is necessary to identify inflammatory-related genes associated with breast cancer to scientifically predict the prognosis. In this study, we focused on investigating how inflammation and its related genes affect the prognosis of breast cancer patients. We conducted a 7 inflammation-related genes risk signature analysis by combining high-throughput data to predict the prognosis of breast cancer patients. And the results demonstrated that our prognostic model could accurately predict the prognosis of breast cancer, which may provide a new ideal and point of reference for the clinical prediction and treatment of breast cancer.

## Materials and Methods

### Patient information and database

We used the GSEA database (http://www.gsea-msigdb.org/) and obtained a total of 200 inflammatory-related genes (IRGs) from the gene set HALLMARK_INFLAMMATORY_RESPONSE. The clinical data, RNA-Seq, immune subtypes, and stemness scores based on DNA-methylation (DNAss) and mRNA (RNAss) were downloaded from the project TCGA-BRCA in the TCGA datasets (https://portal.gdc.cancer.gov/). Of all patient samples in TCGA-BRCA, 1097 cancer samples and 191 para-cancerous samples met the requirement of corresponding complete age, gender, stage, overall survival (OS), and survival status. These qualified samples would be used for subsequent analysis.

### Screening of differential genes and prognostic inflammatory-related DEGs

We used the “DEseq2” package in R software (R version 4.1.3) to screen the differentially expressed genes (DEGs) in cancer and adjacent tissues [23]. (*p* < 0.05; logFC filter > 1.5) were set as the filter conditions. Then we further performed the univariate Cox hazards regression analysis on the obtained IRGs, and generated candidate prognosis-related genes with a significant difference in OS (*p* < 0.05) through the two-sided log-rank tests with the ‘survival’ package in R. The forest plot was utilized to display the *p*-value, 95% CI, and hazard ratio of each variable with the ‘forest plot’ package.

### Establishment and validation of IRGs-based risk assessment model

To determine the value of inflammatory genes in evaluating the prognosis of breast cancer, we used the LASSO-COX univariate regression analysis to build a prognostic model that can predict the risk of patient survival based on candidate IRGs. Then, the Cox regression model was established with the “glmnet” R package [24], and through cross-validation, we successfully avoided the overfitting of the prognostic genes. To measure the value of each IRG in the risk assessment model, we calculated the corresponding coefficients. The risk score is calculated using the following formula:

$$Risk\;Score=\overset{n}{\underset{i=0}{\sum }}coeff_{i}\left(genes\right)*expr_{i}\left(genes\right)$$

‘Genes’ denoted each IRG, including GPR132, IFITM1, IL12B, IL18, IRF7, KCNMB2, and TACR1; “expr” denoted the gene expression level of IRGs normalized by Log2; “coef” for the coefficient of IRG in the univariate Cox regression analysis. Then the risk score for each patient was calculated. According to the median risk scores, breast cancer patients were divided into high-risk and low-risk groups. The prognostic difference between the two groups was analyzed by the Kaplan-Meier survival analysis. To verify the prognostic value of candidate IRGs, the “time ROC” package in R was used to calculate the area under the time-dependent receiver operating characteristic (ROC) curve (AUC) to evaluate prediction efficiency [25]. The higher the area under the curve (AUC) value, the higher the accuracy of the prediction. T-distributed stochastic neighbor embedding (t-SNE) and principal components analysis (PCA) mapping were used to explore the distribution in different groups based on the expression level of genes in the model and measure whether the survival status was well distributed.

### Construction and evaluation of prognostic nomogram

IRGs were selected as an independent prognostic factor for patients with breast cancer by univariate Cox regression analysis (*p* < 0.05). Nomogram can predict the prognosis of cancers and display the results of the risk model precisely [26]. To research the relationship between the IRGs and breast cancer patients, these IRGs were integrated to establish a genomic nomogram by the “rms”, “nomogramEx”, and “regplot” packages in R software and to predict the 1-, 2-, and 3-year survival possibilities of each patient [27]. Besides, we performed the univariate and multivariate Cox Hazards regression analysis to determine whether IRG-based risk scores, along with other possible risk factors such as age and TNM stage, were significant predictors of prognosis in breast cancer.

### Tumor microenvironment characteristics and function enrichment analysis

A gene set enrichment analysis (GSEA) was performed to illuminate the enrichment of high- and low-risk breast cancer groups in terms of immune function. Further, using the single-sample gene set enrichment analysis (ssGSEA) and the relative R package was “GSVA” to quantify the differences between the high- and low-risk groups for the scores of tumor-infiltrating immune cells and immune-related pathway activity [28]. Besides, the Estimation of Stromal and immune cells in Malignant Tumors using the Expression data (ESTIMATE) algorithm was performed to calculate the immune score, stromal score, and ESTIMATE score by the R package “ESTIMATE” [29].

### Drug sensitivity analysis

To clarify the influence of inflammatory genes on drug sensitivity and tolerance in the prognostic model, NCI60 drug response data from the CellMiner tool (https://discover.nci.nih.gov/cellminer) were downloaded. This database included 60 different cell lines derived from 9 malignancies that must be screened when developing new anti-tumor drugs and 262 drugs licensed by the FDA or in clinical trials [30]. The relationship between gene expression and drug sensitivity was utilized by the Pearson correlation test.

### Statistical analysis

All statistical analyses were performed with R software (version 4.1.3) and GraphPad Prism 7. Statistical significance was established at a probability value of *p* < 0.05, and all statistical tests were two-sided. We established a risk prognosis model through the LASSO Cox regression algorithm. And the Kaplan-Meier survival curve was generated by the overall survival rate in the high- and low-risk expression groups. The ROC curve evaluated the accuracy of the prognostic model. The univariate and multivariate Cox regression analyses were used to evaluate the feasibility of the risk score and whether it could be an independent prognostic factor. Based on mRNA expression (RNAss) and DNA methylation pattern (DNAss), the Spearman’s test was used to evaluate the relationship between risk scores and cancer stemness scores, while the Pearson’s test was used to evaluate the correlation between gene expression and drug sensitivity in the model.

## Results

### Identification of prognostic inflammation-related genes

**Figure 1:**
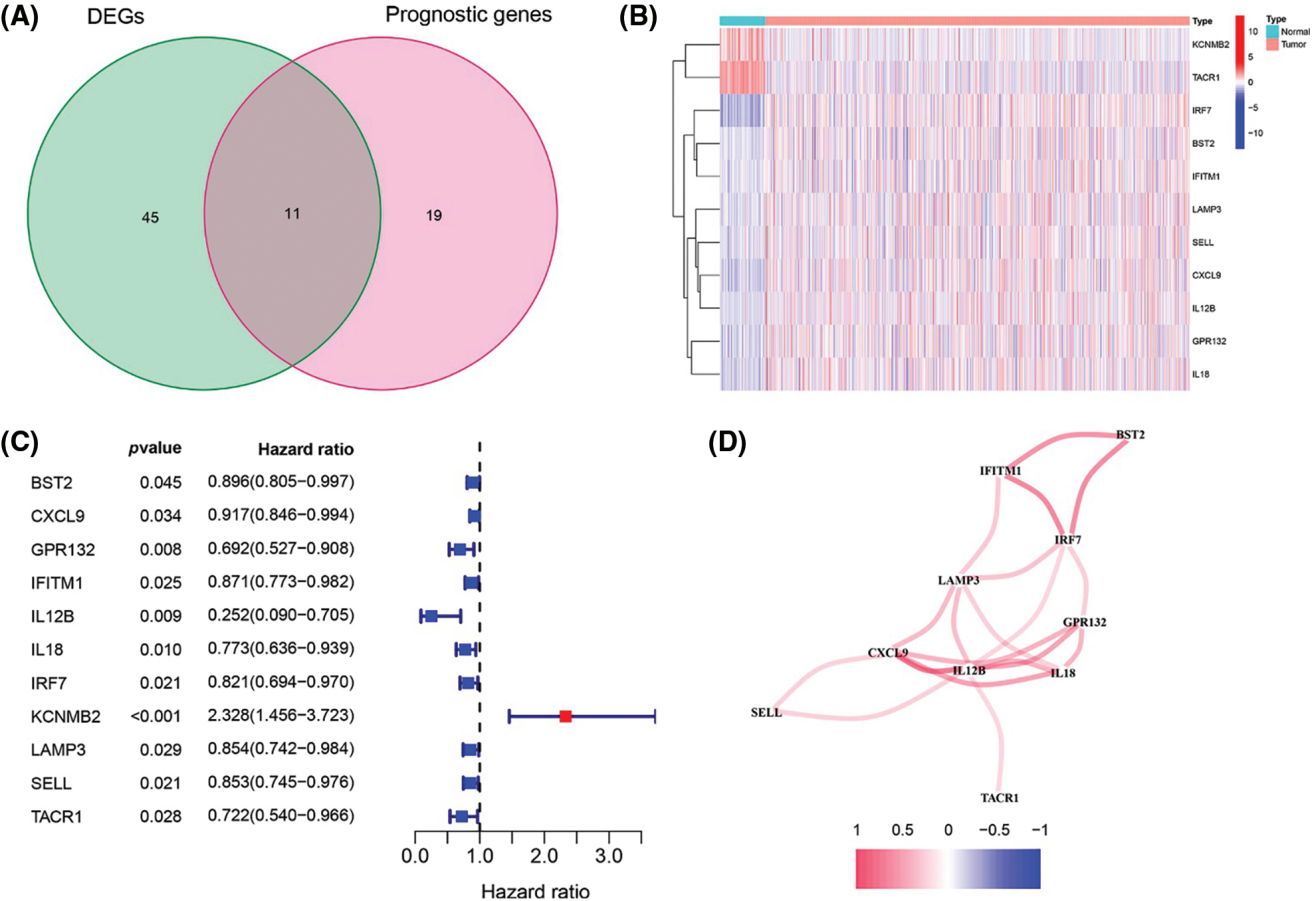
Screening of inflammation-related genes in breast cancer. (A) Venn diagram showed the 11 overlapped genes of DEGs and prognostic genes correlated with the inflammatory responses; (B) Expression heatmap of all inflammatory differential gene expression in breast cancer and normal tissues; (C) Forest map of hazard ratios for 11 prognosis-related inflammatory genes; (D) Correlation analysis of signature genes. Red for a positive correlation; Blue for a negative correlation.

**Figure S1:**
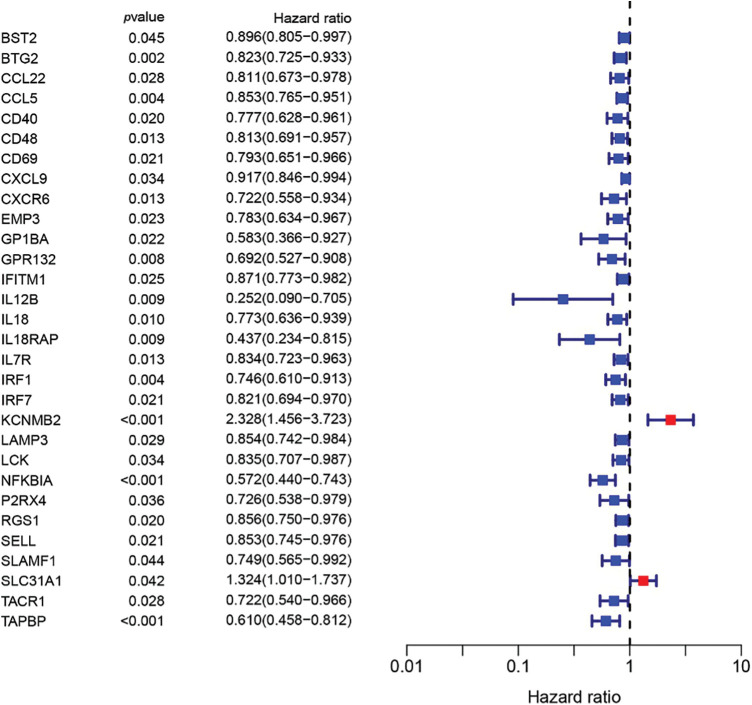
30 inflammation-related genes were related to overall survival.

To build a prognostic prediction model for breast cancer, we obtained the human inflammatory response gene set from the GSEA database, which contained 200 inflammation-related genes (IRGs), and further analyzed it. The expression levels of these inflammatory genes in breast cancer tissues and para-cancerous tissues were obtained from the TCGA database, and 56 differentially expressed inflammatory genes (DEGs) were screened out. A univariate Cox analysis of all the IRGs revealed that 30 of them were related to overall survival with *p* < 0.05 in the TCGA cohort (Suppl. Fig. S1). Then, a total of 11 differently expressed inflammation-related genes were identified by intersecting the 56 DEGs and 30 inflammation-related genes that were displayed in the Venn diagram (Fig. 1A). The expression of 9 upregulated genes (TACR1, IRF7, BST2, IFITM1, LAMP3, SELL, CXCL9, IL12B, IL12B, GPR132, IL18) and 2 downregulated genes (KCNMB2, TACR1) in breast cancer were visualized using a heatmap (Fig. 1B). 10 risk genes (Hazard ratio > 1) and 1 protection gene (Hazard ratio < 1) were found to be associated with breast cancer, according to a univariate Cox regression analysis (Fig. 1C). Almost all of the prognostic signature genes were positively correlated, and the correlation between these genes were displayed in Fig. 1D.

### Construction and validation of a prognostic model in breast cancer

**Figure 2:**
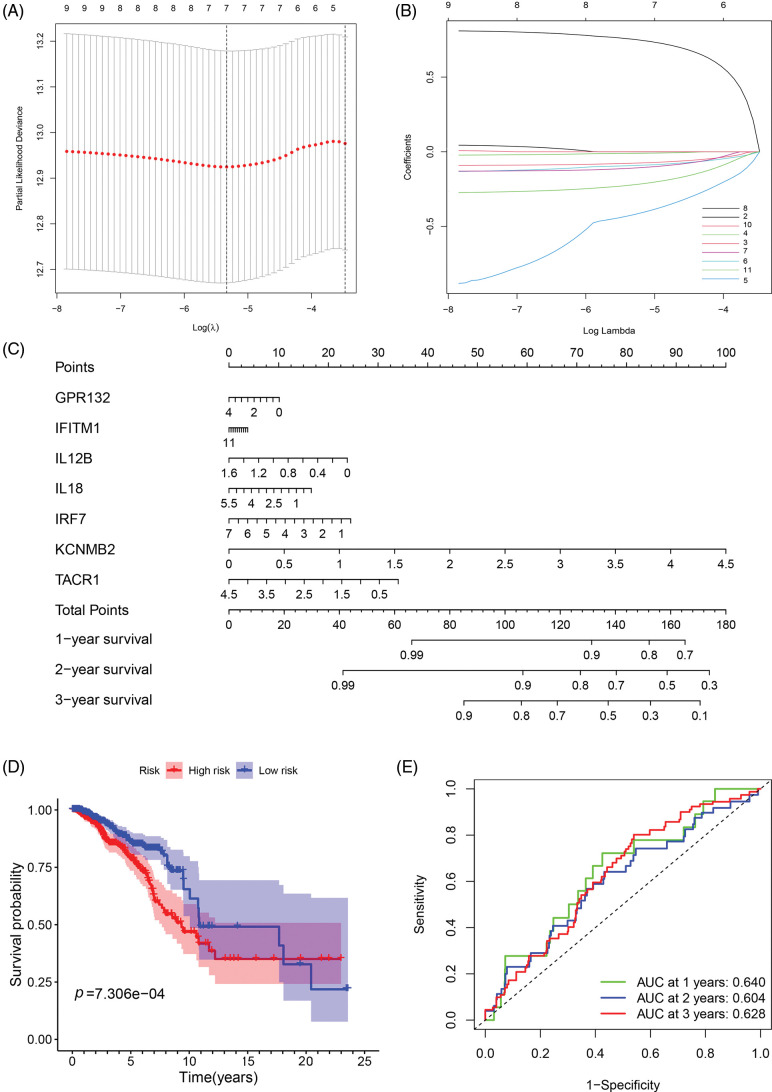
Construction and evaluation of the prognostic model of inflammation-related genes. (A, B) LASSO Cox regression analysis screened 7 differentially expressed inflammatory genes and established a prognostic model; (C) The nomogram for predicting 1-year, 2-year, and 3-year overall survival in breast cancer patients; (D) Survival analysis of breast cancer patients in low- and high-risk groups; (E) ROC curve confirmed the accuracy of the prognostic model.

To investigate the relationship between IRGs and prognosis in breast cancer patients, we constructed a prognostic risk prediction model based on the 11 univariate results through the LASSO algorithm analysis. And the 10-fold cross-validation was utilized to determine the optimal values of the tuning parameter, and the results showed that the 7 IRGs were the most reliable markers (Fig. 2A). There were no zero coefficients in the LASSP coefficients displayed in Fig. 2B. A nomogram was constructed using 7 IRGs, including GPR132, IFITM1, IL12B, IL18, IRF7, KCNMB2, and TACR1, to predict the 1-, 2-, and 3-year survival of breast cancer patients (Fig. 2C). The total score was obtained by accumulating the scores of each gene, and the vertical lines were drawn downward at the corresponding point of the total score to determine the relative survival rates of 1-, 2-, and 3-years. According to the median risk score, patients were classified into low-and high-risk groups. We performed the Kaplan-Meier and ROC analyses to estimate the strength of the IRGs’ signature and compare the differences between the two groups. The KM curves showed a significant difference between the two groups and revealed that patients with higher risk scores tended to have a worse prognosis and shorter survival times (Fig. 2D). Moreover, the areas under the curve of ROC curves (AUCs) at 1, 2, and 3 years were 0.640, 0.604, and 0.628, respectively (Fig. 2E). The results showed the good sensitivity and specificity of the prognostic model.

## Further Validation of the IRGs Risk Prediction Model

**Figure 3:**
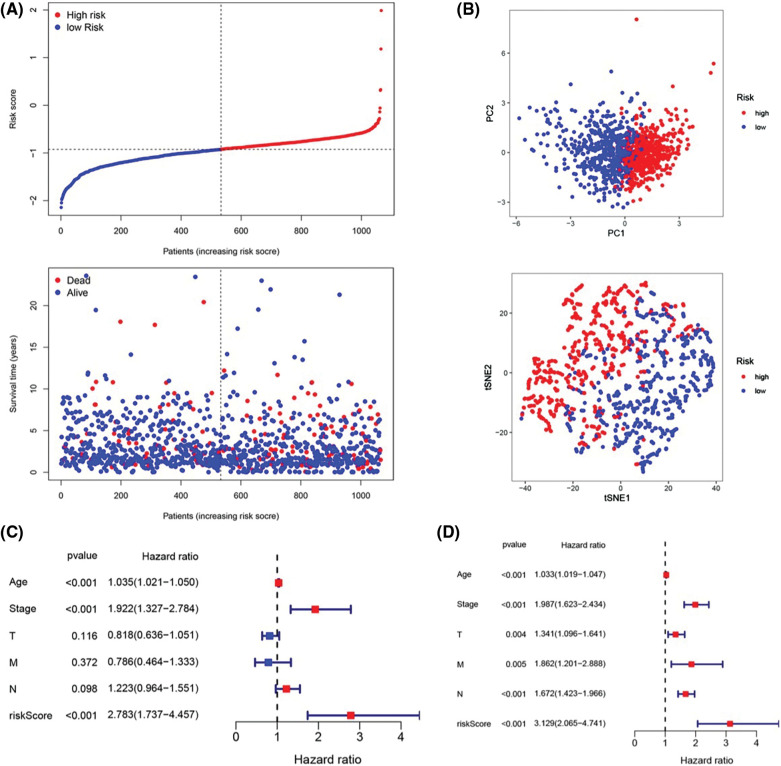
Prognostic value of the candidate prognostic model. (A) Risk curves constructed according to the median of the risk score and the survival status in different risk scores; (B) Feasibility of PCA- and tSNE-based analysis and judgment models; (C) Univariate Cox regression analysis of different clinical characteristics and risk scores; (D) Multivariate Cox regression analysis of different clinical characteristics and risk scores.

To further assess the stratification capabilities of the IRGs risk prediction model, which was constructed based on the TCGA cohort, we divided breast cancers into high- and low-risk groups based on the median risk score. Then we plotted the distribution map of patient survival status and risk score (Fig. 3A). The distribution plot of risk scores revealed more deaths in the high-risk group. Additionally, the scatter plot of the patients’ survival status demonstrated an obviously improved prognosis for the low-risk group. The principal component analysis (PCA) and t-distributed stochastic neighbor embedding (t-SNE) were utilized to confirm the reliable clustering ability of the risk score. The plots demonstrated that breast cancer patients of different risk groups were distributed in two directions, indicating that the expression of 7 genes in the model can effectively classify breast cancer patients into high- and low-risk groups (Fig. 3B). The univariate and multivariate Cox proportional hazard regression analysis was conducted to further explore the independent prognostic value of the inflammation-related genes prognostic risk model. Univariate analysis indicated that age (*p* < 0.001) and pathological (stage) (*p* < 0.001) were significantly correlated with overall survival. Additionally, further multivariate analysis revealed a significant correlation between age (*p* < 0.001), pathological (stage) (*p* < 0.001), lymph node status (N) (*p* < 0.001), and overall survival (Figs. 3C and 3D). The hazard ratio (HR) of the risk score and 95% confidence interval (CI) were 2.783 and 1.737–4.457 in univariate Cox regression analysis (*p* < 0.01), and 3.129 and 2.065–4.741 in multivariate Cox regression analysis (*p* < 0.01). These results suggested that the risk score of the model was a powerful independent predictor of the prognosis in breast cancer patients.

### Evaluation of tumor microenvironment based on IRGs

**Figure 4:**
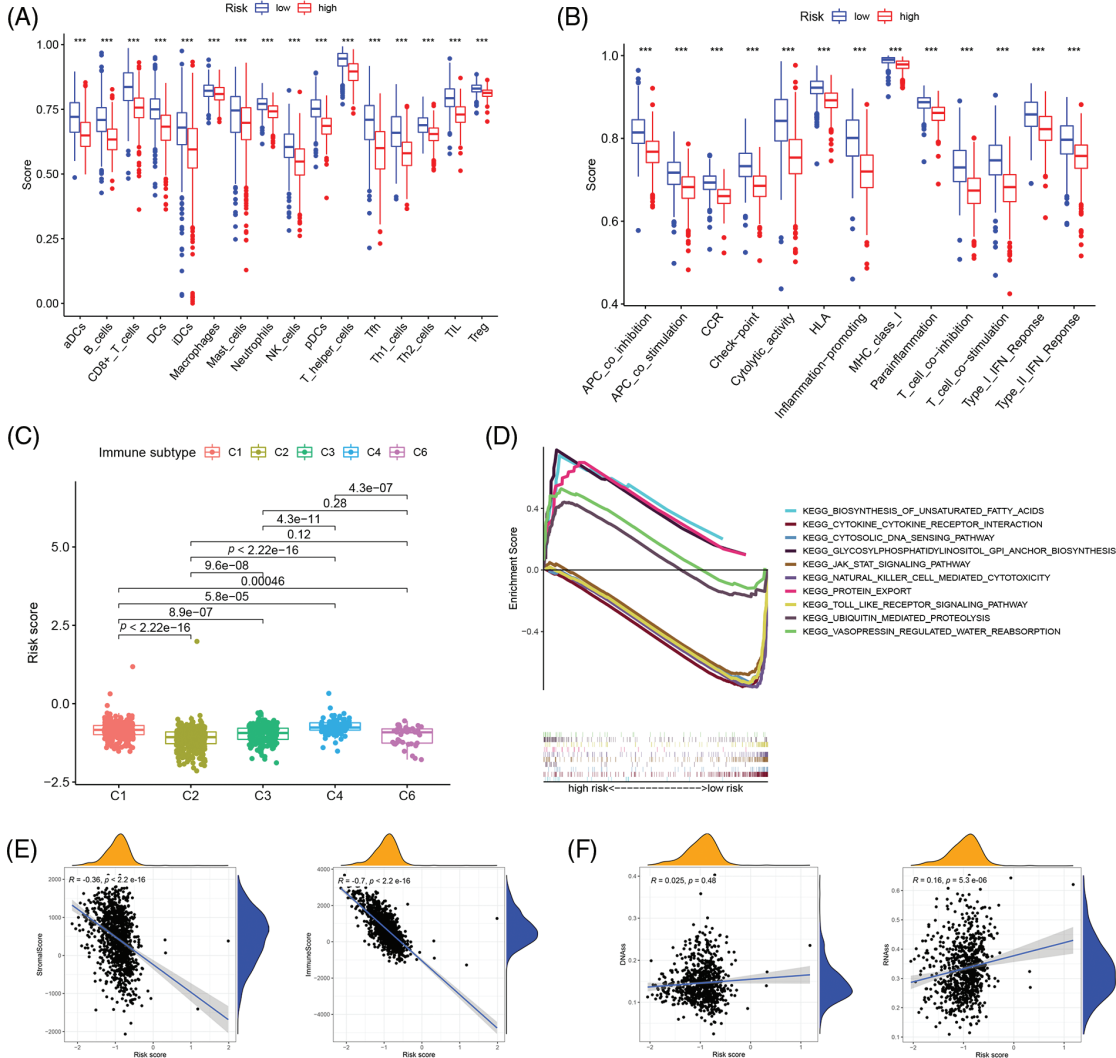
Correlation analysis of immune infiltration patterns based on IRGs. (A) The difference of immune cell subsets in low- and high- risk groups of an inflammation-related prognosis model; (B) The difference of immune function and pathway in the low- and high-risk groups of inflammation-related prognosis models; (C) The difference between breast cancer patients with different risk scores and immune classification; (D) KEGG analysis showing functional enrichment in risk groups; (E) Scatterplot of correlation between the immune cell score, stromal cell and risk score. (F) Scatterplot of correlation between the DNAss, RNAss and risk score (***p < 0.001).

The single sample gene set enrichment analysis (ssGSEA) was used to quantify 16 immune cell subsets and 13 immune-related functions to clarify the correlation between the risk score and immune status. In the TCGA cohort, the results showed that the immune cell infiltration status was often high in the low-risk groups, and the immune-related pathway was also increased in those groups (Figs. 4A and 4B). From the findings, we could conclude that the immune response may be more active in the low-risk group than in the high-risk group, and the poor prognosis of breast cancer patients in the high-risk group may be correlated with negative immune regulation. According to the immunophenotyping distribution of various tumor sample types in the TCGA database, the levels of risk scores for 5 immune types were displayed through the one-way ANOVA analysis (Fig. 4C). Then, we further researched the intrinsic function of IRGs and the associated signal transduction pathway through GSEA, the results demonstrated that the pathways were differentially enriched between low- and high-risk groups (Fig. 4D). Immune status and stromal cells play an important role in the tumor microenvironment. We performed a correlation analysis of the risk score and tumor microenvironment to better understand the impact of the tumor microenvironment on the prognosis of breast cancer patients. The results demonstrated that the risk score was negatively correlated with immune cell infiltration (*p* < 0.001, *R* = −0.36) and stromal cells (*p* < 0.001, *R* = −0.7) (Fig. 4E). And also, spearman correlation tests were performed based on stem cell score (DNAss) and stem cell score (RNAss) to explore the association between risk scores and cancer stemness scores, and the results revealed a positive correlation between RNAss and risk score (*p* < 0.05, *R* = 0.16) (Fig. 4F). As a result, there may be a strong relationship between the risk score of the prognostic model and the activity of cancer stem cells.

### The relationship between IRGs and drug sensitivity

**Figure 5:**
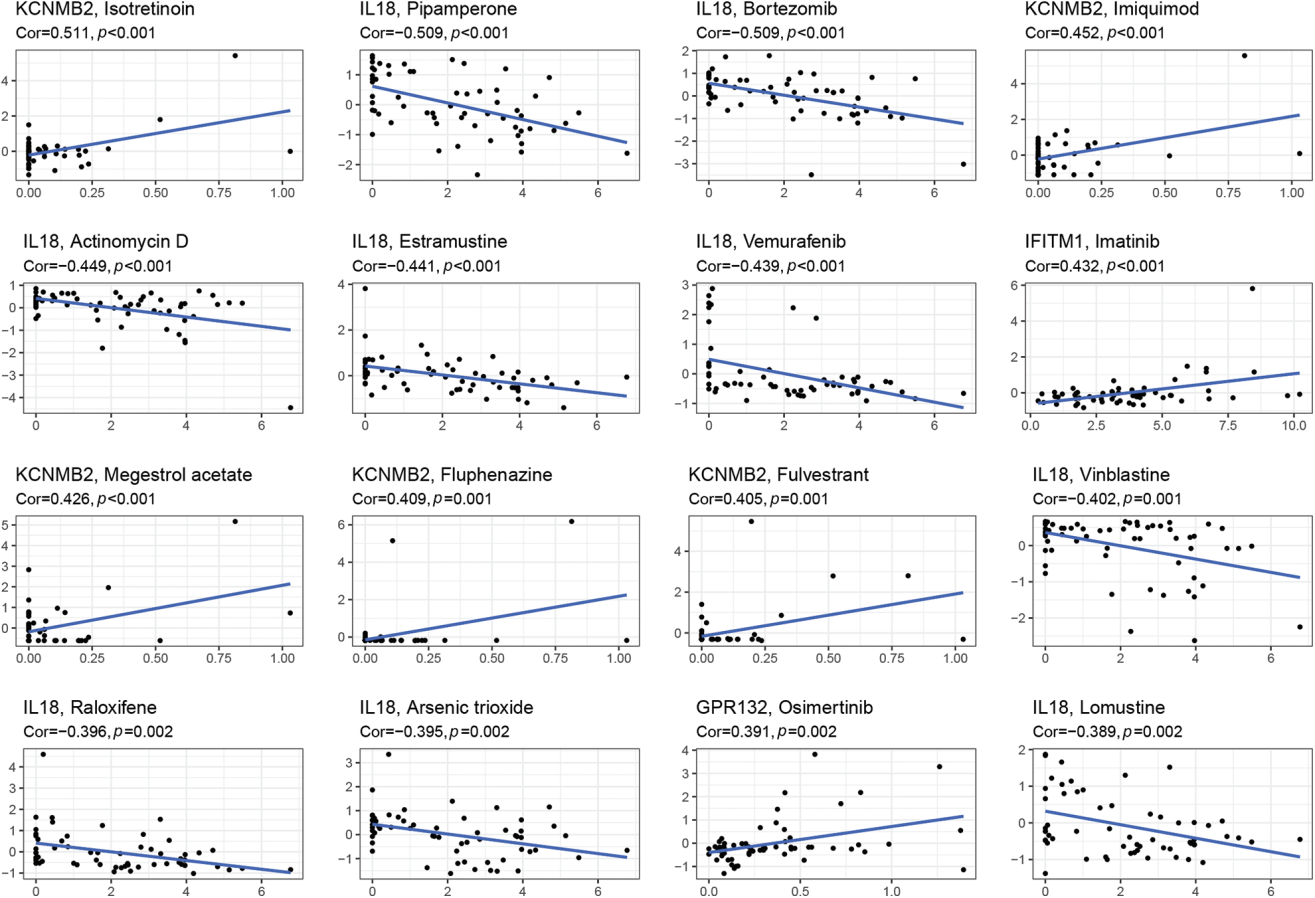
Gene-drug sensitivity analysis based on the CellMiner database and screened out the top 16 drugs with the highest correlation with gene expression in inflammation-related prognosis models.

Through the 60 diverse human cancer cell lines (NCI-60) database, which was assessed via the CellMiner interface (https://discover.nci.nih.gov/cellminer), we explored the effect of IRGs on drug sensitivity and obtained 16 drugs with statistically significant differences (Fig. 5). The results demonstrated that the expression of KCNMB2 was positively correlated with the sensitivity of Isotretinoin, Imuiquimod, Megestrol acetate, Fluphenazine, and Fulvestrant. It represented that the higher the expression of KCNMB2, the stronger the sensitivity to the above-mentioned drugs. The expression of IL-18 was negatively correlated with the sensitivity of Pipamperone, Bortezomib, Actinomycin D, Estramustine, Vemurafenib, Vinblastine, Raloxifene, Arsenic trioxide, and Lomustine. In addition, the expression of IFITM1 has a positive correlation with Imatinib, and GPR has a negative correlation with Osimertinib. The investigation demonstrated that our risk score calculation model could effectively predict the sensitivity of cancer cells to these drugs and could lead to more precise drug use in clinical settings.

## Discussion

In recent years, the comprehensive treatment of breast cancer has achieved, significant advances, and the survival of most patients has greatly improved, but some breast cancer patients still face the risk of recurrence and metastasis [5,31]. At present, clinical, pathological, and molecular are used to determine therapeutic strategies and evaluate the prognosis of patients [32,33]. But due to the significant heterogeneity of breast cancer, these factors still cannot be used to precisely evaluate the prognosis of patients. Therefore, new biomarkers that could accurately predict the prognosis of breast cancer are urgently needed.

Cancer-associated systemic inflammation is strongly related to poor disease outcome in patients [34,35]. The inflammatory microenvironment can provide favorable conditions for the expansion and mutation of tumor cells [36]. A prior study has demonstrated that 8 inflammatory response-related genes have been linked to prognosis and immunological status in hepatocellular carcinoma, and inhibiting these genes may be a treatment option [37]. Recent studies have demonstrated a close relationship between some inflammatory cells and inflammatory factors and breast cancer [18,38,39]. Additionally, there has been a gradual increase in research on prognostic risk prediction models for breast cancer. Only a few studies have developed inflammation-based prognostic markers, despite the fact that inflammation has been found to play a role in breast cancer. Therefore, it is meaningful and innovative to research the prognosis of breast cancer patients from the perspective of inflammation-related factors. In our study, a prognostic signature of 7 genes associated with inflammation precisely identified the survival of breast cancer patients during robustness evaluation. Inflammation-related genes could serve as possible biomarkers and potential therapeutic targets for patients with breast cancer.

It is a very effective bioinformatics strategy to establish predictive models using data from TCGA and GEO databases that sequence the whole genomes of breast cancer patients. In this study, we established an inflammatory risk model to predict the prognosis of breast cancer. Firstly, we identified 200 inflammatory-related genes from the gene set of the GSEA database, and then 11 prognosis-related genes were screened out using R software. The univariate Cox regression analysis revealed that 11 IRGs all showed prognostic values. LASSO algorithm analysis was used to construct an inflammatory risk prediction model based on the expression of prognostic IRGs and survival. 7 robust IRGS were identified by 10-fold cross-validation analysis, including GPR132, IFITM1, IL12B, IL18, IRF7, KCNMB2, and TACR1. We used the IRGs to build a classifier. Patients were divided into low- and high-risk groups according to their risk scores. The KM survival analyses showed that patients with higher risk scores tended to have a poorer prognosis and survival. The ROC demonstrated that the prognostic model had good sensitivity and specificity. And the risk score could be regarded as a powerful predictor through analyses such as PCA, t-SNE, and univariate and multivariate Cox regression.

Tumor inflammation is closely associated with immune cell infiltration in the tumor microenvironment, which contributes to the immunotherapy response. We compared tumor infiltration between the two risk groups in this study. We found that patients in the low-risk group had higher proportions of immune cells such as CD8^+^ T cells, B cells, DCs, Macrophages, and so on. These immune cells could contribute to anti-tumor immunity and were positively associated with the prognosis of breast cancer [40,41]. Finally, we performed tumor microenvironment characteristics and drug sensitivity analysis of these IRGs and found there was a significant correlation. It indicated that we could make accurate and effective decisions when selecting drugs in clinical trials.

In conclusion, we established an inflammatory risk model to predict the prognosis of breast cancer based on TCGA database. Firstly, differentially expressed inflammatory genes were identified and constructed into a prognostic model using LASSO. The bioinformatics analysis, which included ROC, risk score, Kaplan Meier analysis, univariate and multivariate cox regression analysis, and more, proved the excellent ability to predict the prognosis of gene signatures based on inflammation. Finally, we examined the features of the tumor microenvironment and drug sensitivity of these differentially expressed genes. In summary, it was discovered that a higher risk score was strongly associated with a worse prognosis for breast cancer, which could be useful to clinicians in helping them make accurate and effective decisions.

## Data Availability

The datasets presented in this study can be found in online repositories, the accessible link of the database is as follows: GSEA database (http://www.gsea-msigdb.org/), TCGA datasets (https://portal.gdc.cancer.gov/). Other data that support the findings of this study are available on request from the corresponding author.
